# Effect of Salmeterol-Fluticasone Combination and Tiotropium on Clinical and Physiological Improvement of Bronchial Anthracofibrosis: a Double Blind Randomized, Cross Over, Placebo Controlled, Clinical Trial

**Published:** 2018-03

**Authors:** Majid Mirsadraee, Shadi Ghaffari, Parisa Saeedi

**Affiliations:** 1 Department of Internal Medicine, Faculty of Medicine, Islamic Azad University, Mashhad, Iran; 2 Research Department of Kavosh High School, Mashhad, Iran; 3 Department of Urology, Akbar Hospital, Mashhad University of Medical Sciences, Mashhad, Iran.

**Keywords:** Anthracofibrosis, Anthracosis, Treatment, Salmeterol, Fluticasone, Tiotropium

## Abstract

**Background:**

Bronchial anthracosis is the black discoloration of bronchial mucosa that exhibits similar manifestations to Chronic Obstructive Pulmonary Disease ( COPD). The etiology of this obstructive lung disease has not been elucidated and standard therapy for this disease has not been introduced in the literature. The objective of this study is to determine the efficacy of the salmeterol-fluticasone inhaler and tiotropium as two safe treatments of obstructive lung disease for the treatment of symptomatic subjects of anthracofibrosis of the lung.

**Materials and Methods:**

Twenty anthracofibrosis subjects who suffered from dyspnea were enrolled in this three-phase, cross over, placebo-controlled clinical trial. The primary outcome variable was quality of life (evaluated with the CAT questionnaire). Clinical findings and spirometry were the secondary outcome variables. Both of these drugs were delivered by an inhaler and were made identically by the reference manufacturer. Salmeterol-fluticasone was prescribed with a spacer and tiotropium by its special device, and the method of utilization was taught to the patients.

**Results:**

Twenty anthracofibrosis subjects were enrolled in this three-phase, five-month course of treatment with either salmeterol-fluticasone or tiotropium inhalers. The response to therapy was not good; neither for salmeterol-fluticasone nor for tiotropium in the short course of the treatment. However, the overall results of 5 months of therapy with both of the drugs have shown improvement in 57% of the subjects. The most prominent results were found in the CAT score [25.1±5.54 before the trial, which decreased to 19.2±5.14 (Z score=2.7, P=0.007)] and clinical findings especially sputum, chest pain, and wheezing (81, 94 and 92% before the trial and 50, 56, 54% after the trial, respectively). Neither clinical findings nor spirometry was able to predict a good response to salmeterol-fluticasone or tiotropium.

**Conclusion:**

The combination of salmeterol-fluticasone and tiotropium inhaler was able to improve the clinical findings of symptomatic anthracofibrosis patients.

## INTRODUCTION

Bronchial anthracosis is the black discoloration of bronchial mucosa and is an old disease that is being increasingly reported in Asia, especially in rural areas (1,2). Sometimes this is an accidental finding during bronchoscopy, but in a more severe form called “anthracofibrosis” it causes obliteration and deformity of bronchial lumen. This form is important due to its clinical resemblance to Chronic Obstructive Pulmonary Disease (COPD) and lung cancer (3), and its association with tuberculosis (4). Moreover, our experience has shown that episodes of severe attacks of dyspnea and wheezy chest superimpose to the previous slowly progressive disease (5); very similar to acute exacerbation of COPD, albeit without a history of cigarette smoking. During bronchoscopy, several of these subjects showed localized involvement that required no therapy, while several of them showed extensive involvement that caused clinical symptoms by itself. Pulmonary Function Tests (PFT) showed obstructive lung disease with normal Diffusing capacity of the lungs for carbon monoxide ( DLCO) (6), especially in subjects suffering from wide spread involvement of airways. Presently, no established treatment for anthracosis has been introduced, but the experiences of Iranian lung specialists have shown that the disease could be controlled with a combination of Long-Acting Beta2-Agonists (LABA) and Inhaled Corticosteroid (ICS). However, because of the similarity of this disease with COPD, a role for tiotropium has been anticipated.

The objective of this study is to determine the efficacy of the salmeterol-fluticasone combination and tiotropium for the treatment of symptomatic subjects of anthracofibrosis of the lung.

## MATERIALS AND METHODS

A double blind, randomized, three-way, crossover, clinical trial registered in the Iranian Clinical Trial Registry (No: IRCT201404072695N5) conducted in a tertiary subspecialty pulmonary clinic.

According to the frequency of anthracofibrosis in our previous study (11%) (5), twenty subjects were enrolled in this study.

Anthracofibrosis subjects who exhibited a new acute attack of respiratory symptoms (either cough or dyspnea), wheezing in chest auscultation and FEV1 less than 80% in spirometry (either restrictive or obstructive pattern) were enrolled in this study. Definite diagnosis of anthracofibrosis was made before the study was started by bronchoscopy.

Exclusion criteria were as follow: having another accompanying pulmonary disease in addition to anthracofibrosis such as asthma, tuberculosis, COPD, Idiopathic Pulmonary Fibrosis (IPF), tuberculosis, or cancer.

A Persian language informed consent was designed and given to each patient along with oral explanations.

### Outcomes and variables

A questionnaire for clinical findings including dyspnea, cough, sputum, chest pain, and wheezing in the physical examination, spirometry parameters including FEV1, FVC, FEV1/FVC, MMEF, MEF50%, and quality of life (evaluated with the CAT questionnaire) were completed at the beginning of the trial and were checked after each phase. Clinical findings were evaluated as positive or negative and staging was not performed.

The CAT questionnaire score was our most valuable result and considered as the primary end point.

Drugs and their strength: 1) Salmeterol-fluticasone combination administered in a metered dose inhaler containing 25 μg of salmeterol and 250 μg of fluticasone in each puff (Seroflo^®^, Cipla Co., Goa, India) in two puffs, twice daily by a spacer. 2) Tiotropium (KP-Tiova^®^, Cipla Co., Goa, India) administered with its special device for inhalation (Revolizer^®^) in doses of 18μg once daily. The method of using these drugs were discussed and demonstrated to the patients.

**Figure 1.**
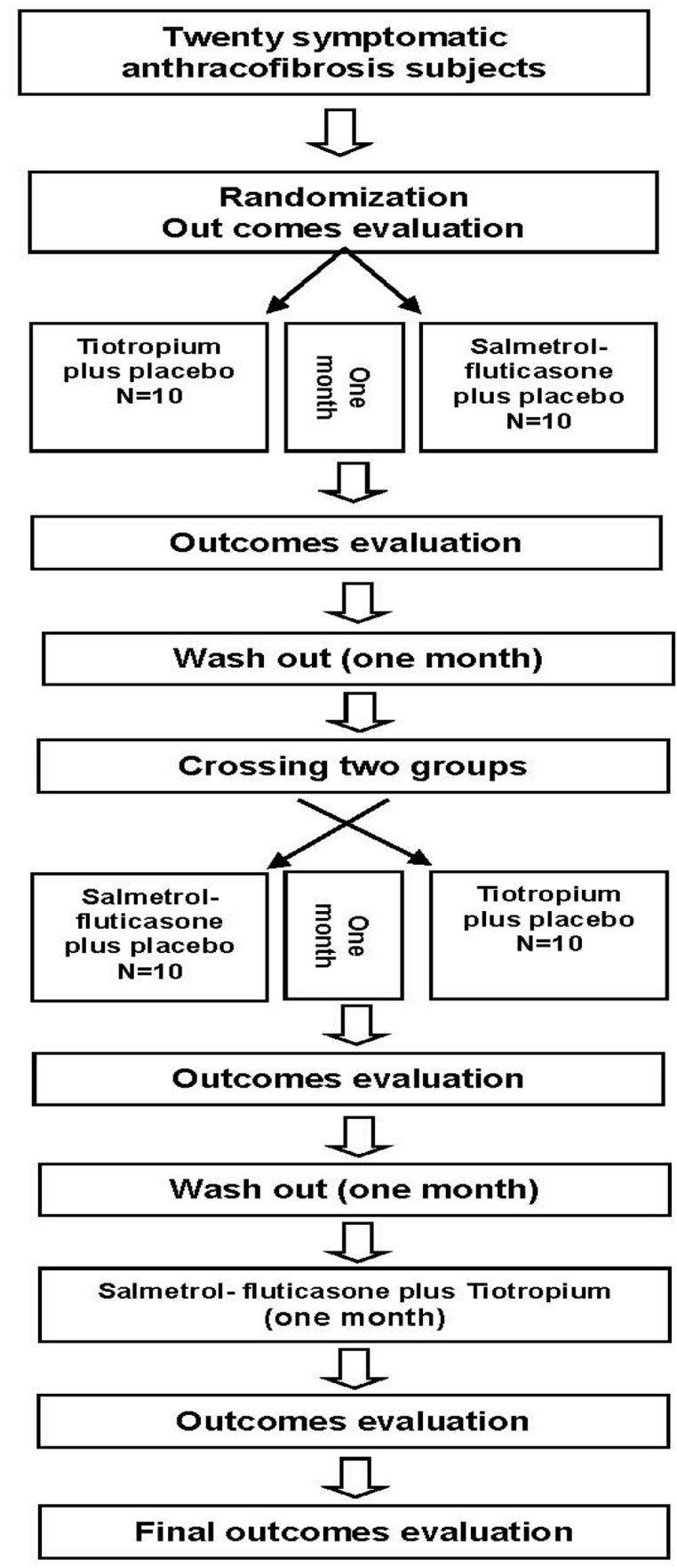
Study protocol in a three phase, cross over, clinical trial about effect of slametrol-fluticason and/or tiotropium on symptomatic anthracofibrosis patients.

Randomization and random allocation: Placebos for the salmeterol-fluticasone inhaler and tiotropium were obtained from the producer of the drugs (Cipla Co., Goa, India) and were completely similar in appearance to the originals. The drugs were coded before the study and then a pharmacist, blinded to the situation of the subjects, distributed the drugs to them. The physician or any of the patients did not know the type of drugs they were started on. The drugs were randomly distributed to the two groups using a computational random number generator (SPSS software). Anthracofibrosis subjects were divided into two groups randomly based on study protocol (Figure 1).

### First phase

For the first group (10 subjects), the salmeterol-fluticasone combination was prescribed and a placebo in place of tiotropium for one month. The second group (10 subjects) was prescribed tiotropium and a placebo for the salmeterol-fluticasone combination. After one month, subjects were reassessed by three tools to measure outcome variables as mentioned above, and then all of the subjects were administered a placebo for a drug washout.

### Second phase

Next, the group's subjects were crossed over to the other group and administered the other drug and placebo for one month. Afterward, they were evaluated again for outcome variables and were administered the placebo for drug washout.

### Third phase

The subjects in both groups were combined (20 subjects) and received salmeterol-fluticasone and tiotropium together for one month. At the end of this phase outcome variables were evaluated for the fourth time and the subjects were questioned regarding the side effects of the drugs. At the end of this phase, the study was completed.

### Statistical analysis

According to the frequency of anthracofibrosis in our previous study (11%), 20 subjects were proven to show 80% power for detecting 10% change in FEV1 or the CAT score. The results of similar treatment protocol of phases 1 and 2 were accumulated. The results of the study were compared before and after the study by the McNemar test for clinical findings, Wilcoxon signed rank test was used for the CAT questionnaire test results, and the paired t test for spirometry parameters. This method of analysis was also used to evaluate the results between the first visit and the outcomes at the end of the third phase. The results of the outcome variable were compared between subjects who showed good response and poor response. In this analysis, the student t test and chi square were used.

## RESULTS

Twenty anthracofibrosis subjects, whose diagnosis were proven by bronchoscopy and suffered from respiratory symptoms, were enrolled in this study. Bronchoscopic findings showed that anthracofibrosis was localized in some lobar bronchi in five (25%) subjects and it was diffuse in 15 (75%) subjects. One subject showed associated lung fibrosis in addition to anthracofibrosis. These 20 subjects completed one-month courses of salmeterol-fluticasone combination/placebo and tiotropium/placebo therapy sequentially. From these two groups, two subjects improved and did not continue with the third phase of the salmeterol-fluticasone combination/tiotropium therapy.

### Demographic data

Mean age was 73±12.3 (42–89) years. Most of the subjects were female (18 subjects) and all of them were homemakers. One male subject worked as a driver and another was a businessman. Sixty percent of subjects lived in the city, although all of them have lived most of their life in rural areas. Frequency of water pipe smoking was reported in 60% of female subjects. Opium addiction was noted in three subjects including one of the male subjects. In addition, exposure to wood smoke during baking was reported in 13 female subjects (72%), which is significantly higher than the male subjects who did not report participating in baking activities (X^2^=4.1, P=0.02). Cigarette smoking was not reported in any subject.

Mean duration of the disease was 88.1±10.3 months (1–508). The course of the disease was chronic, persistent in 16/18 (89%) of the subjects, a new onset in one subject (5.6%), and intermittent with acute exacerbation in another subject (5.6%).

Computed tomography was performed in eleven subjects. Lymph node high attenuation (calcified like lesion) was observed in 5/11 (45%) and a mass with or without high attenuation in 8/10 (80%).

### Effects of the salmeterol-fluticasone inhaler on anthracofibrosis

Table 1 shows the clinical findings of the anthracofibrosis subjects who received salmeterol-fluticasone and a placebo of tiotropium. Among these five major clinical findings, dyspnea was the most frequent and wheezing in the physical examination showed the best improvement, although none of the changes after the treatment were statistically significant.

**Table 1.** Effects of the salmeterol-fluticasone inhaler on clinical findings of anthracofibrosis subjects

	Before trial	Salmeterol-fluticasone	Tiotropium	Mixed	Final
Cough	**15/19(79%)**	**16/19 (84%)**	**12/18(66%)**	**13/17 (76%)**	**11/14 (78%)**
Dyspnea	**19/19 (100%)**	**17/17(90%)**	**16/17 (94%)**	**15/17(88)**	**14/16(88%)**
Wheezing	**14/18 (78%)**	**10/18 (56%)**	**13/17 (88%)**	**10/16 (62%)**	7/13 (54%)^*^
Sputum	**13/19(68%)**	**14/19 (74%)**	**8/18 (44%)**	**10/17 (76%)**	8/16 (50%)^*^
Chest pain	**15/18 (83%)**	10/18 (56%)^*^	**8/17 (47%)**	**14/17 (82%)**	9/16 (56%)^*^

* Significant difference evaluated by the McNemar test.

**Figure 2.**
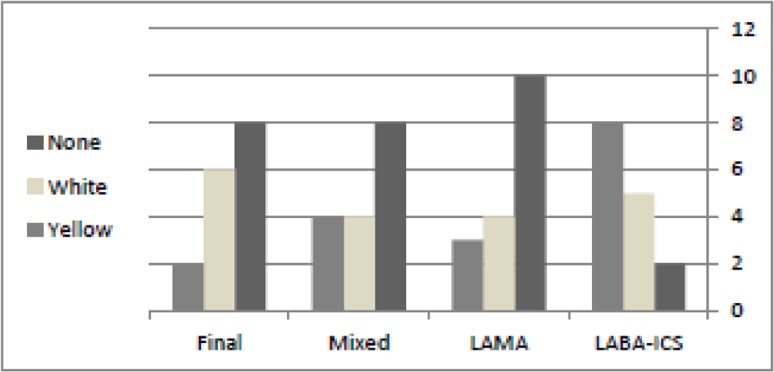
Frequency of sputum appearance before and after treatment with slameterol-fluticasone or tiotropium in subjects suffering from anthracofibrosis.

From nineteen subjects who responded to the question about the color of sputum, four subjects (21%) reported whitish sputum, nine subjects (47%) yellow, and six subjects (31%) reported transparent sputum (31%). After the treatment, yellow sputum decreased to two subjects (13%) and white sputum increased to eight subjects (53%), in which the differences were significant (X^2^=12, P=0.01) (Figure 2).

Evaluation of the CAT questionnaire showed improvement in some parameters especially step tolerance (P=0.02), disability during work, and sleep quality (Table 2). Total CAT score as the cumulative result of the CAT questionnaire showed severe disability and low quality of life, which improved non-significantly with treatment (Table 2).

**Table 2.** Effect of tiotropium on quality of life assessed by the CAT questionnaire on anthracofibrosis subjects

	**Before trial**	**Salmeterol-fluticasone**	**Tiotropium**	**Mixed**	**Final**
**Cough**	2.1±1.25	2.5±1.71	1.8±1.29	2±1.32	1.8±1.32
**Sputum**	1.9±1.71	2.3±1.86	1.2±1.2^*^	1.3±1.27	1.2±1.29^*^
**Dyspnea**	3.8±1.14	3.5±1.17	3.5±1.2	3.2±1.21^*^	3.0±1.15^*^
**Step tolerance**	4.0±0.82	3.4±0.84^*^	3.5±0.9	3.5±1.06	1.6±1.50^*^
**Work tolerance**	3.8±0.89	3.2±0.78^*^	3.6±0.84	2.9±1.24	2.6±1.20^*^
**Outside activity**	3.3±0.87	3.4±0.77	3.4±0.6	2.9±1.14^*^	2.8±0.98^*^
**Sleep**	2.6 ±1.53	3.4±0.84^*^	2±1.5	2.7±1.5	1.6±1.5
**Weakness**	3.3±0.73	2.8±1.07	3±0.8	2.8±0.92	2.7±1.06
**Total score**	25.1±4.83	23.6±7.12	22.3±5.09	21.7±6.41	19.2±5.14^*^

* Z evaluated by the Wilcoxon signed rank test

**Figure 3.**
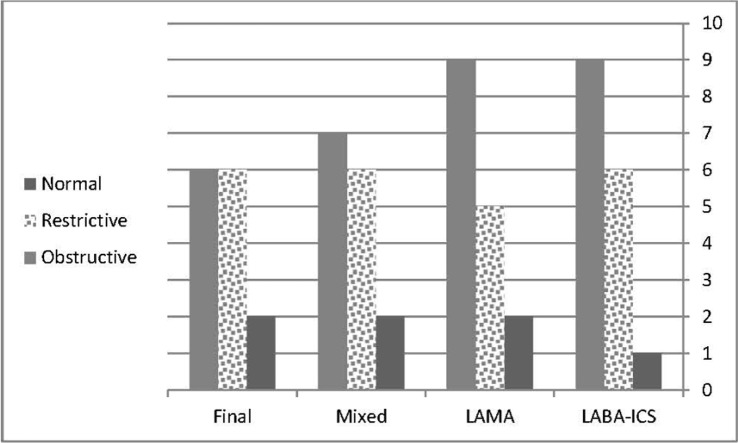
Classification and interpretation on spirometry before and after treatment with slameterol-fluticasone or tiotropium in subjects suffering from anthracofibrosis

Spirometry parameters showed moderate mixed restrictive and obstructive type impairment (Table 3). Treatment with salmeterol-fluticasone was able to improve the small airway obstruction as shown by significant improvement of FEF25-75 and FEF25-75/FVC (Table 3). Restrictive pattern was the predominant pattern of the spirometry before and after the trial (Figure 3).

**Table 3.** Effects of the salmeterol-fluticasone inhaler on spirometry results of anthracofibrosis subjects

	**Before trial**	**Salmeterol-fluticasone**	**Tiotropium**	**Mixed**	**Final**
**FVC (L)**	1.2±0.62	1.1±0.39	1.3±0.5	1.3±0.53	1.2±0.51
**FVC percent**	61±18.2	62.0±17.0	67.5±22.09	63.8±19.12	63.7±17.32
**FEV1 (L)**	0.9±0.33	1.03±0.31	1.01±0.37	1.07±0.40	1.05±0.40
**FEV1 percent**	66.5±21.9	70.1±22.4	67.9±21.4	65.9±20.4	69±19.4
**FEV1/FVC**	86.7±8.5	90.5±9.19	85.6±11.6	79.1±25.67	85.2±13.07
**FEV1/VC (%)**	87.5±20.4	92.5±15.7	82.7±14.9^*^	72.3±24.3	86.8±16.61
**FEF25-75 (L/S)**	1.0±0.33	1.14±0.38^*^	0.98±0.47	1.07±0.42	1.27±0.67^*^
**FEF25-75 percent**	44.6±15.7	50.5±22.9	44.8±20.15	48.8±28.3^*^	60.9±26.5^*^
**FEF25-75/FVC**	0.87±0.21	1.0±0.29^*^	0.83±0.44	0.83±0.21	1.1±0.69^*^

### Effects of tiotropium on anthracofibrosis

Table 1 shows the clinical findings of anthracofibrosis subjects who received the tiotropium inhaler and a placebo and table 2 shows the clinical findings and quality of life evaluated by the CAT questionnaire in these subjects. Although some improvement could be found in some parameters such as cough, sputum, and sleep quality, the statistical analysis did not show significant differences. Sputum type was mostly whitish in appearance, but after treatment with tiotropium, it changed to no sputum (Figure 2). Spirometry findings are shown in table 3, in which no significant changes were revealed with the treatment of tiotropium. The cumulated interpretation of spirometry according to the parameters and flow volume curve before the trial were normal in one subject (6%), obstructive in six (37%), and restrictive in nine (57%). After the trial there was not any significant change and only two subjects' results changed from the restrictive to obstructive pattern; the others remained the same (Figure 3).

### Effects of the combination of tiotropium and salmeterolfluticasone inhaler on anthracofibrosis

Improvement in some clinical findings were evident by using the tiotropium inhaler and the salmeterol-fluticasone in our anthracofibrosis subjects, especially in those with sputum and wheezing (41 and 31% improvement, respectively) (Table 1), but the statistical analysis did not show significant changes. Figure 2 shows the different types of sputum in these subjects and it shows non-significant decrement of whitish and mucoid sputum toward subjects without sputum (X^2^=12, P =0.21). On the other hand, analysis of the CAT questionnaire results showed significant improvement in dyspnea and outside activity (P-value 0.01 and 0.05, respectively).

Other parameters of the CAT score, including total score, showed some improvement, although not significant (Table 2). Spirometry analysis also did not show significant changes before and after treatment with the tiotropium and salmeterol-fluticasone inhaler (Table 3). Final classification of spirometry results have shown significant decrement of the obstructive pattern (X^2^= 26.8, P=0.002) (Figure 3).

### Cumulated results of the three phases

Seventeen subjects finished five months of the three-phase course of the trial and three subjects exited the trial by their own decision. The whole course of the study was two years and one of the subjects died from her previous cardiac disease. All of the 17 subjects were female and homemakers. Ten subjects (58.8%) reported substantial improvement in their disease. The results of the treatment from the beginning of the final phase are described in this section. Comparison of clinical findings before and after the five-month course of treatment showed non-significant resolution of cough and dyspnea and significant improvement and disappearance of chest pain, sputum, and wheezing in the physical examination (Table 1).

Sputum type was present in 13/15 (81%) subjects (Table 1) before the trial and sputum was whitish in 8/16 (50%) subjects and yellow in 6/16 (37%) subjects (Figure 2).

After the complete course of the study, sputum disappeared in five subjects (31%) and the color of sputum changed significantly to a whitish color in six (75%) subjects and in two (25%) subjects it turned to yellow (Figure 2) (X^2^=20, P=0.01).

Assessment of quality of life by the CAT questionnaire also showed improvement of quality of life. The total CAT score was 25.1±5.54 before the trial, which decreased to 19.2±5.14 (Z score=2.7, P= 0.007) (Table 2). The changes during the trial were not significant for cough, sleep quality, and weakness, but the remaining parameters were improved significantly (Table 2).

Analysis of spirometry parameters showed no significant differences between FEV1, FVC, FEV1/FVC, and FV1/VC (Table 3), but mid flow parameters such as FEF25-75, FEF25-75% predicted and FEF25-75/FVC as representative of small airway disease, showed significant improvement (Table 3).

Figure 3 shows the final interpretation of spirometry. It seems that the restrictive pattern decreased after treatment with the complete course, but statistical analysis was not significant (McNemar p-value= 0.55).

### Predicting factors for good response

Demographic data, clinical findings, CAT score, and spirometry results were compared between the two groups of responders and non-responders to determine the best predicting factors for good response to treatment.

Tables 4 and 5 show the most suitable demographic, occupational, clinical, radiological, and physiological (spirometry) findings that could help determine a predictive factor for good response to long term bronchodilator with or without inhaled corticosteroid.

**Table 4.** Comparison of demographic, occupational, clinical, and radiological parameters between anthracofibrosis subjects who improved or did not improve with the long-term bronchodilator with or without inhaled corticosteroid

	**Improved**	**Not improved**	**X2**	**P value**	**Odds ratio**	**95% CI**
**Sex**	10/10 (100%)	7/7 (100%)	-	-	-	-
**Chronic course**	8/9 (89%)	4/5 (80%)	2.3	0.3	0.5	0.02–10
**Baking**	10/10 (100%)	5/7 (71%)	3.2	0.07	3	1.4–6.1
**Smoking**	4/10 (40%)	2/5 (40%)	0.23	0.62	1.6	0.2–13.2
**Rural/Urban**	5/5	4/3	0.08	0.77	0.75	0.1–5.2
**Cough**	7/9 (78%)	6/6 (100%)	1.5	o.21	1.85	1.12–3.07
**Dyspnea**	10/10 (100%)	7/7(100%)	-	1	-	-
**Wheezing**	7/8 (87%)	6/6 (100%)	0.8	0.36	1.8	1.12–3.07
**Sputum**	8/10 (80%)	6/7 (86%)	0.09	0.76	0.66	0.04–9.18
**Chest pain**	9/10 (90%)	7/7 (100%)	0.74	0.38	1.77	1.15–2.73
**LN**	3/6 (50%)	3/2 (60%)	0.3	0.8	0.8	0.2–3
**Mass**	8/12 (66%)	4/12 (33%)	0.66	0.41	4	0.13–119

**Table 5.** Comparison of demographic and clinical assessment by the CAT questionnaire and physiological (spirometry) parameters between anthracofibrosis subjects who improved or did not improve with the long-term bronchodilator with or without inhaled corticosteroid

	**Improved**	**Not improved**	**t test**	**P value**
**Age (y)**	77.4±9.1	70±14.7	1.16	0.26
**Time of disease(M)**	110±144	65±58	0.77	0.45
**Cough CAT**	2.1±1.8	3.1±1.3	1.3	0.21
**Sputum CAT**	2.5±1.77	3±1.41	0.61	0.54
**Dyspnea CAT**	4±1.41	4.14±0.88	0.23	0.81
**Step CAT**	4.1±1.1	4±1	0.19	0.85
**Work CAT**	3.8±0.91	3.1±1.46	1.14	0.27
**Outside activity CAT**	3.4±0.69	3.2±0.48	0.37	0.71
**Sleep CAT**	2.3±1.6	2.7±1.7	0.48	0.63
**Weakness CAT**	3.4±0.96	2.8±1.06	1.09	0.29
**Total CAT**	25.6±7.39	26.2±5.5	0.2	0.83
**FVC (L)**	1.03±0.41	1.75±0.96	1.9	0.06
**FVC percent**	60±14.7	68±21.58	0.9	0.37
**FEV1 (L)**	0.86±0.34	1.08±0.45	1.15	0.26
**FEV1 percent**	61.8±20.52	69.5±16.59	0.8	0.42
**FEV1/FVC**	84±17.17	87.16±13.79	0.36	0.72
**FEV1/VC (%)**	92.9±24.3	90.8±15.74	0.17	0.86
**FEF25-75 (L/S)**	0.99±.0.6	1.04±0.43	0.15	0.87
**FEF25-75 percent**	44.3±12.9	45.1±13.82	0.11	0.9
**FEF25-75/FVC**	0.97±0.72	0.72±0.36	1.04	0.31

Comparison of these risk factors did not show any significance for any of them, but the odds ratio and 95% confidence interval showed a role for baking, cough, wheezing, and chest pain for predicting a good response. Furthermore, the type of sputum in improved subjects was equally whitish and yellowish in color in both groups; therefore, it was not a predicting factor for improvement.

## DISCUSSION

In this three-phase, cross over, clinical trial, we enrolled 20 non-treated anthracofibrosis subjects and managed their disease with a salmeterol-fluticasone inhaler, tiotropium or both. The results of the study have shown that the short course of treatment with either the salmeterol-fluticasone inhaler or tiotropium did not show significant benefits, but long-term assessment of the whole study showed improvement in clinical findings and quality of life in 58% of subjects. No side effects were reported during the course of the trial and one of the subjects died from an unrelated heart disease. Baking, coughing, wheezing, and chest pain were the most important predicting factors for a good response to the therapy. Anthracofibrosis of the lung is an old disease in the human population (5); however, no definite etiology or treatment has been introduced. Biomass exposure is the most confirmed risk factor associated with anthracosis and anthracofibrosis. In the present research, biomass exposure and baking was reported in 72% of subjects. In a previous study done in our country, baking was associated with a four-fold elevated risk of anthracosis (7). In Western countries, as biomass use has decreased considerably, occupational exposure to coal has remained the most important risk of acquiring native anthracosis (8).

However, as the origin of an anthracotic nodule in the vesicles of bronchial macrophage is undefined, none of these studies were able to introduce an effective treatment targeting the pathophysiology of the disease. Most comprehensive reviews recommend avoiding exposure to organic and in-organic dusts (9), but this strategy will not relieve the clinical symptoms and illness of anthracofibrosis patients.

Treatment of associated tuberculosis usually makes a considerable clinical and radiological improvement (10,11). Decisions about starting anti-tuberculosis therapy are quiet easy, as the diagnosis of anthracofibrosis requires bronchoscopy and during bronchoscopy enough samples for the diagnosis of tuberculosis can be accumulated (12).

We should assume that all subjects of anthracofibrosis may not be associated with tuberculosis (13), making anti-tuberculosis therapy a good approach for the treatment of anthracofibrosis. During bronchoscopy severe inflammation and erythema in the area not involved by anthracosis may be seen. Traditionally oral corticosteroids are used in our region and one study recommends them in anthracofibrosis (14). However, because of its side effects we should consider it as an emergency drug for severe exacerbations. On the other hand, inhaled corticosteroids have low side effects; therefore, they could be a good substitute for oral corticosteroids. In this research, fluticasone was selected as the inhaled corticosteroid.

Since there is no method presently to remove an anthracotic nodule from the bronchial mucosa, authors of this research and many other physicians in our region prescribe the long acting bronchodilator for the treatment of anthracofibrosis. The rational of selecting this treatment is adapted from COPD. We should remember the influence of biomass in the formation of both COPD and anthracofibrosis. Therefore, choosing long acting bronchodilators might be prudent in anthracofibrosis such as COPD. In this study, long acting beta2-agonist (salmeterol) and long acting anti-muscarinic agent (tiotropium) were chosen for the treatment and were compared with each other. Salmeterol was combined with inhaled corticosteroids to better cover asthma-like bronchial disease. All of these drugs are safe and are available in low-income countries. The results of this research have shown that beneficial effects of these drugs cannot be shown in the short course of therapy, but long-term usage is helpful. Due to the low mortality and morbidity rate of this disease (1,5) in comparison to COPD, we recommend using these drugs for the long term; usually more than three months, especially during the winter season and to start with one drug and add another one if an appropriate response is not seen. We also recommend another research to compare the effect of LABA alone with the combination of LABA-ICS.

In conclusion, the long acting bronchodilator including long acting beta2-agonist and long acting muscarinic antagonist with or without inhaled corticosteroids are safe and effective drugs for the treatment of symptomatic bronchial anthracofibrosis.

### Conflict of interest

The authors of this article did not receive any grant from any drug company and the provider of placebo delivered the placebo's free of charge and did not get involved in any part of design and analysis of this study.
